# Origins and Stepwise Expansion of R2R3-MYB Transcription Factors for the Terrestrial Adaptation of Plants

**DOI:** 10.3389/fpls.2020.575360

**Published:** 2020-12-23

**Authors:** Xiaojun Chang, Shupeng Xie, Lanlan Wei, Zhaolian Lu, Zhong-Hua Chen, Fei Chen, Zhongxiong Lai, Zhenguo Lin, Liangsheng Zhang

**Affiliations:** ^1^College of Horticulture, Fujian Provincial Key Laboratory of Haixia Applied Plant Systems Biology, Fujian Agriculture and Forestry University, Fuzhou, China; ^2^Suihua Branch of Heilongjiang Academy of Agricultural Sciences, Suihua, China; ^3^Department of Biology, Saint Louis University, St. Louis, MO, United States; ^4^School of Science, Western Sydney University, Penrith, NSW, Australia; ^5^Hawkesbury Institute for the Environment, Western Sydney University, Penrith, NSW, Australia; ^6^College of Horticulture, Faculty of Plant Science, Nanjing Agricultural University, Nanjing, China; ^7^Genomics and Genetic Engineering Laboratory of Ornamental Plants, College of Agriculture and Biotechnology, Zhejiang University, Hangzhou, China

**Keywords:** R2R3-MYB transcription factors, embryophytes, chlorophytes, charophytes, land plant adaptation

## Abstract

The R2R3-MYB transcription factors play critical roles in various processes in embryophytes (land plants). Here, we identified genes encoding R2R3-MYB proteins from rhodophytes, glaucophytes, Chromista, chlorophytes, charophytes, and embryophytes. We classified the R2R3-MYB genes into three subgroups (I, II, and III) based on their evolutionary history and gene structure. The subgroup I is the most ancient group that includes members from all plant lineages. The subgroup II was formed before the divergence of charophytes and embryophytes. The subgroup III genes form a monophyletic group and only comprise members from land plants with conserved exon–intron structure. Each subgroup was further divided into multiple clades. The subgroup I can be divided into I-A, I-B, I-C, and I-D. The I-A, I-B, and I-C are the most basal clades that have originated before the divergence of Archaeplastida. The I-D with the II and III subgroups form a monophyletic group, containing only green plants. The II and III subgroups form another monophyletic group with Streptophyta only. Once on land, the subgroup III genes have experienced two rounds of major expansions. The first round occurred before the origin of land plants, and the second round occurred after the divergence of land plants. Due to significant gene expansion, the subgroup III genes have become the predominant group of R2R3-MYBs in land plants. The highly unbalanced pattern of birth and death evolution of R2R3-MYB genes indicates their important roles in the successful adaptation and massive radiation of land plants to occupy a multitude of terrestrial environments.

## Introduction

It is widely accepted that transcription factors (TFs) play vital roles in all aspects of plant life (Riechmann and Ratcliffe, 2000; Singh et al., 2002). TF genes account for a large proportion of protein-coding genes in plant genomes (Zhang et al., 2020a). For instance, 5% of protein-coding genes (∼1500) in the *Arabidopsis* genome encode transcription factors (Riechmann et al., 2000), and about 45% of these are from families specific to plants. *Arabidopsis* transcription factors do not share significant similarity with those of the other kingdoms beyond the conserved DNA binding domains, many of which have been arranged in combinations that are specific to plants (Riechmann et al., 2000). The increasing availability of genome and transcriptomic data of plants have enabled better understandings of the functional roles of TFs and their contributions to the evolution of green plants and their adaptation to terrestrial habitats (Hori et al., 2014; Bowman et al., 2017; Nishiyama et al., 2018; Cheng et al., 2019).

Among plant TF families, the MYB gene family is one of the most extensively studied groups due to their family size and functional significance (Dubos et al., 2010). MYB proteins contain one to four tandemly located MYB domains, which were categorized into R1, R2, or R3 types (Jin and Martin, 1999; Feller et al., 2011). According to the number and type of present MYB domains, MYB genes were classified into four families: 1R-MYB, R2R3-MYB (or 2R-MYB), R1R2R3-MYB (or 3R-MYB), and 4R-MYB (Dubos et al., 2010; Xu et al., 2018). The R2R3-MYB proteins, which have two adjacently located repeats (R2 and R3), are the predominant family of MYB proteins in plants. Many R2R3-MYB genes are required for cell differentiation and other developmental processes, response to various environmental stresses, and secondary metabolism (Dubos et al., 2010).

The origin and early diversification of land plants from charophyte algae, which consist of only a few cells, is one of the most important events of the evolution of the plant kingdom, as it involved many unprecedented evolutionary innovations (Kenrick and Crane, 1997). These evolutionary processes enabled the subsequent diversification and success of land plants, with a series of morphological and physiological innovations, such as the stem with vascular tissue to transport fluid and nutrients, epidermal structure (stomata) for respiratory gas exchange, as well as leaves and roots (Kenrick and Crane, 1997). Understanding how land plants evolved from ancestral algal species remains a major challenge in plant biology (Bowman et al., 2017; Nishiyama et al., 2018). Many plant-specific protein families, including the R2R3-MYB family, were believed to have played vital roles in facilitating this transition to land (Feller et al., 2011; Zhao et al., 2019). Previous studies suggested MYB genes had polyphyletic origins (Dubos et al., 2010; Bowman et al., 2017; Jiang and Rao, 2020; Liang et al., 2020), but the origin and evolutionary history of R2R3-MYBs are still not well understood due to the limited coverage of species in some lineages of green plants in these studies.

Charophytes (streptophyte algae) represent an intermediate group between chlorophytes and land plants (embryophytes). Therefore, it is necessary to include MYB genes from charophytes for a better understanding of the early evolution of R2R3-MYB genes (Bowman et al., 2017). The genomes of chlorophyte species *Klebsormidium flaccidum* (re-identified as *Klebsormidium nitens*) and *Chara braunii* have been recently sequenced (Hori et al., 2014; Nishiyama et al., 2018). The transcriptomes of many charophyte, chlorophyte, glaucophyte, Chromista, and rhodophyte species have been generated by the One Thousand Plant Transcriptomes (1KP) project^1^. The availability of these new genomic and transcriptomic data makes it possible to conduct a more comprehensive phylogenetic inference of the R2R3-MYB family. In this study, we identified R2R3-MYB genes from over 200 plant species that represent all six major plant lineages (rhodophytes, glaucophytes, Chromista, chlorophytes, charophytes, and embryophytes), with a focus on charophytes, representing the most comprehensive survey of R2R3-MYB genes to date. We conducted systematic phylogenetic inferences and studies of gene exon–intron structure. Based on our results, we classified R2R3-MYB genes into three subgroups (I, II, and III), representing their evolutionary origins. Our further interrogation of the evolutionary history of each subgroup revealed two major expansion events during the evolution of subgroup III. We speculated these expansion events might have contributed to the successful adaptation of land plants to terrestrial environments. In summary, our study provides a more informative evolutionary history of the R2R3-MYB gene family, and improves our understanding of the roles of R2R3-MYB genes in the evolution of green plants.

## Materials and Methods

### Data Sources

The complete genome and predicted proteome or predicted transcript sequences data of plant species were obtained from NCBI, JGI Genome Portal^2^, Phytozome^3^, and Genome Warehouse^4^. The proteome of *K. nitens* was obtained from *Klebsormidium* genome project (Hori et al., 2014). The proteome of *Chara braunii* was obtained from *Chara* genome project (Nishiyama et al., 2018). The proteomes of the other five charophytes were obtained from Beijing Genome Institutes (Cheng et al., 2019; Wang et al., 2020). For the proteome datasets, if two or more protein sequences at the same locus were identical where they overlapped, we selected the longest sequence. Furthermore, we identified R2R3-MYB homologous genes from transcriptomic data of 191 species from charophyte, chlorophyte, glaucophyte, Chromista, rhodophyte, and liverworts, which were obtained from the 1KP project^5^.

### Homolog Searches

The hidden Markov model-based HMMER program (3.0^6^) was used to identify all proteins containing MYB domains. The MYB domain (PF00249: Myb_DNA-binding_ls.hmm) in Pfam 23.0 (Finn et al., 2010) was used to perform local searches in the downloaded proteome datasets. The proteins meeting the following criteria were considered as valid R2R3-MYB proteins and included in this study: (1) Only two MYB domains are detected in a protein sequence; (2) the minimum bit score of domain match is 35; and (3) the two MYB domains are separated by fewer than 20 amino acids (Du et al., 2015). Therefore, a total number of 541 R2R3-MYB proteins were identified from the 33 examined species. The list of 541 genes and the locations of MYB domains is provided in Supplementary Table S1. Amino acid sequences of the R2R3-MYB proteins are provided as Supplementary Files 1–3.

### Phylogenetic Analyses

The amino acid sequences of the two MYB domains were retrieved from each R2R3-MYB protein for multiple sequence alignment, which was carried out using MUSCLE (v3.8.31) (Edgar, 2004). The phylogenetic trees of R2R3-MYB were reconstructed using maximum likelihood (ML). The best-fit substitution model was inferred by using ProtTest (Abascal et al., 2005). The best model is LG + G, according to Bayesian information criterion (BIC) and corrected Akaike information criterion (AICc). ML tree was inferred using PhyML 3.0 (Guindon et al., 2010), with the LG substitution model and gamma correction for rate heterogeneity across sites. The shape parameter α of the gamma distribution was estimated directly from the data. ML phylogenies were also inferred using IQ-TREE (Nguyen et al., 2015) (parameters: –TEST -bb 1000). For each reconstruction, the best model was selected using the –TEST parameter, and 1000 ultrafast-bootstraps were computed. ML phylogenies were also inferred using FastTree^7^. Among them, IQ-TREE is used to verify the accuracy of the topology. Although IQ-TREE is also an ML method, it is not the same as the strategy used in PhyML 3.0. Fasttree is used to calculate a large number of genes.

## Results

### Three Subgroups of the R2R3-MYB Gene Family

We first identified R2R3-MYB genes from all algae with complete-sequenced genomes and annotations of protein-coding genes, which included four red algae (rhodophytes), 16 green algae (chlorophytes), and six charophytes (charophytes) (Table 1). Seven land plant species that represent the major lineages of embryophytes were selected for this study, including *Marchantia polymorpha* (liverwort), *Physcomitrella patens* (moss), *Sphagnum fallax* (moss), *Selaginella moellendorffii* (lycophyte), *Amborella trichopoda* (basal angiosperms), *Oryza sativa*, and *Arabidopsis thaliana* (Table 1). Consistent with previous studies (Dubos et al., 2010; Du et al., 2015), we defined an R2R3-MYB TF as a protein containing two tandemly located MYB domains (see section “Materials and Methods”). Because the sequences of R2R3-MYB proteins outside the MYB domains are highly diverse, only the R2 and R3 domains were used for reconstruction of phylogenetic trees.

**TABLE 1:** Number of R2R3-MYB genes in examined species.

Taxonomic group	Species name	Subgroup			Total
		I	II	III	
Rhodophyta (Red algae)	*Chondrus crispus*	3	0	0	3
	*Cyanidioschyzon merolae*	3	0	0	3
	*Galdieria sulphuraria*	3	0	0	3
	*Porphyra umbilicalis*	3	0	0	3
Chlorophyta (Green algae)	*Auxenochlorella protothecoides*	3	0	0	3
	*Bathycoccus prasinos*	5	0	0	5
	*Chlamydomonas eustigma*	8	0	0	8
	*Chlamydomonas reinhardtii*	8	0	0	8
	*Chlorella variabilis*	5	0	0	5
	*Coccomyxa subellipsoidea*	4	0	0	4
	*Dunaliella salina*	4	0	0	4
	*Gonium pectorale*	4	0	0	4
	*Helicosporidium* sp.	1	0	0	1
	*Micromonas pusilla*	4	0	0	4
	*Micromonas commoda*	7	0	0	7
	*Monoraphidium neglectum*	8	0	0	8
	*Ostreococcus lucimarinus*	4	0	0	4
	*Ostreococcus tauri*	4	0	0	4
	*Tetrabaena socialis*	7	0	0	7
	*Volvox carteri*	11	0	0	11
Charophyta (Green algae)	*Chlorokybus* sp.	6	0	0	6
	*Mesostigma viride*	2	0	0	2
	*Klebsormidium nitens*	14	3	0	17
	*Coleochaete scutata*	6	3	0	9
	*Spirotaenia* sp.	3	4	0	7
	*Chara braunii*	4	4	0	8
	*Entransia fimbriata*	1	0	0	1
Embryophyta (Liverwort)	*Marchantia polymorpha*	10	4	7	21
Embryophyta (Moss)	*Physcomitrella patens*	14	3	31	48
Embryophyta (Moss)	*Sphagnum fallax*	9	5	19	33
Embryophyta (Lycophyte)	*Selaginella moellendorffii*	8	4	4	16
Embryophyta (Angiosperm)	*Amborella trichopoda*	10	4	37	51
Embryophyta (Monocot)	*Oryza sativa*	18	7	80	105
Embryophyta (Eudicot)	*Camellia sinensis*	16	7	81	104
Embryophyta (Eudicot)	*Arabidopsis thaliana*	24	9	93	126

**FIGURE 1:**
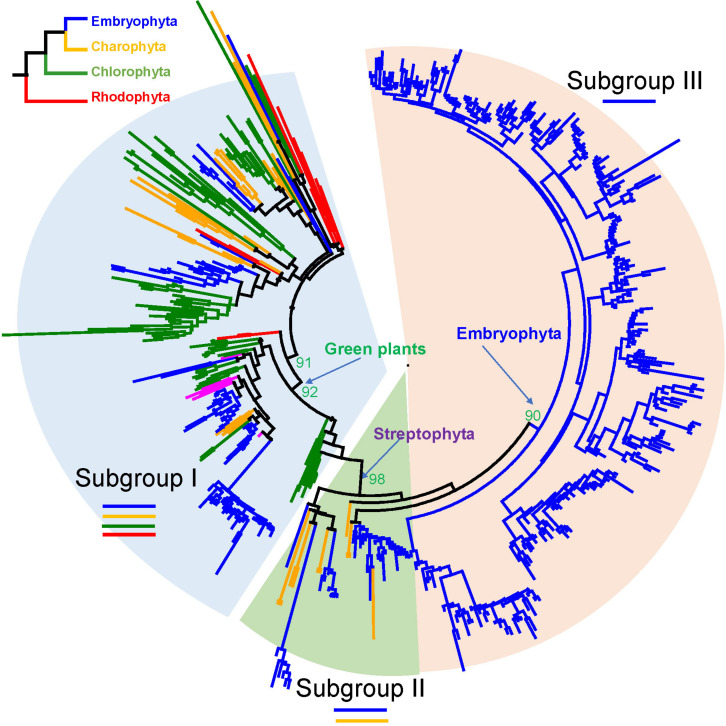
Phylogenetic tree of R2R3-MYB gene family in plants. This phylogenetic tree was reconstructed using 102 sites in the R2 and R3 domains by maximum likelihood (ML) method using PhyML. The evolutionary relationships of the four major plant lineages, rhodophytes, chlorophytes, charophytes, and embryophytes, which are represented by different branch colors, are illustrated in the top left corner.

**FIGURE 2:**
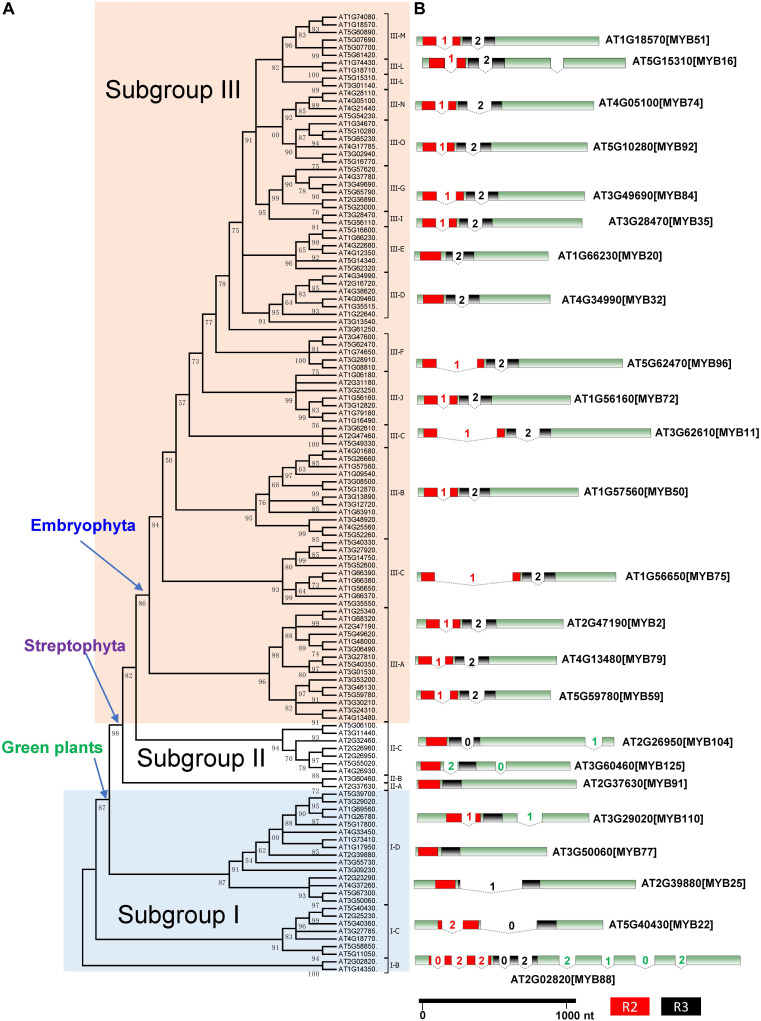
Phylogenetic tree and intron–exon structure of the R2R3-MYB genes in *Arabidopsis thaliana.* **(A)** The maximum likelihood (ML) tree of 126 R2R3-MYB genes from *A. thaliana*. Subgroups I, II, and III refer to different subgroup. **(B)** The intron–exon structure of representative R2R3-MYB genes from each clade. The boxes indicate the protein-coding exons and are drawn to scale. The introns are represented by angled lines, their lengths being proportional to intron length. The number in each intron indicates its phase. The R2 MYB domain is shown as a red box while the R3 MYB domain is shown as a black box.

We built an ML tree based on 541 R2R3-MYB proteins from the 33 representative species (see section “Materials and Methods” and Figure 1). Based on the tree topology and taxonomic distribution of clades, we divided the R2R3-MYB gene family into three subgroups: I, II, and III (Figure 1 and Supplementary Figures S1, S2). The three subgroups can be further divided into 22 well-supported clades: four in subgroup I (I-A to I-D), three in subgroup II (II-A to II-C), and 15 in subgroup III (III-A to III-O) (Figures 1, 2).

To further examine the classification and evolution of R2R3-MYB genes, we conducted more extensive phylogenetic analyses of R2R3-MYB family by including additional species. Specifically, we identified and included R2R3-MYB homologous sequences from 191 species of charophyte, chlorophyte, glaucophyte, Chromista, and rhodophyte based on transcriptomic data obtained from the 1KP project (Supplementary Table S1). We used 3R-MYB genes and CDC5 genes in our phylogenetic analysis to infer the origin of R2R3-MYB genes. The CDC5 genes have diverged from the R2R3-MYB genes before the divergence of eukaryotes (Du et al., 2015), and thus were used as outgroup. The rooted tree (Supplementary Figure S3) further supported the presence of three subgroups of the R2R3-MYB gene family. In addition, it confirmed that subgroup I is the most basal group in the R2R3-MYB family. To test the robustness of the classification of the three subgroups, we constructed another phylogenetic tree using R2R3-MYB genes only from *Arabidopsis*, and the tree topology supports the presence of three subgroups (Figure 2).

In subgroup I, I-A, I-B, and I-C are the only groups that contain members from all Archaeplastida lineages: rhodophytes, chlorophytes, charophytes, and embryophytes (Supplementary Figure S3). They form the most basal clades in the phylogenetic tree, resembling the earliest diverged groups in the subgroup I. I-D form a monophyletic calde with the II and III subgroups, containing only green plants. The subgroup II only consists of genes from land plants and charophyte algae (Supplementary Figure S3). The subgroups II and III together formed a well-supported monophyletic group. The subgroup III genes were only found in land plants and form a well-supported monophyletic group.

### Conservation of Intron–Exon Structures Among Subgroup III Members in Land Plants

The similarities in exon–intron organization among genes can be used as evidence to support their common origin (Sanchez et al., 2003). To obtain additional evidence to support the evolutionary classification of the R2R3-MYB gene family, we examined the intron–exon structure of its members from several representative species. We observed distinct patterns of intron–exon structure between the three subgroups (Figure 2 and Supplementary Figures S4, S5). In *Arabidopsis*, 24 R2R3-MYB genes belong to subgroup I and 9 of them are in subgroup II. The 24 subgroup I genes are distributed into four clades (I-B to I-D), which have different numbers, positions, and phases of introns (Figure 2 and Supplementary Figure S4). The numbers of introns ranged from 0 in clade I-D to nine in clade I-B. Some genes in clade I-C and I-D have two introns, but their positions and phases are different. Clades II-A, II-B, and II-C, contain genes with one or two introns, with different positions and phases. In contrast, the number, position, and phases of introns of the genes in subgroup III are highly conserved, and most of the subgroup III genes contain two introns (phase 1 and phase 2), each at the same location among its members (Figure 2). The phase 1 intron was lost in a small number of subgroup III genes, such as AT4G34990 and AT1G66230. The genes in subgroup III of other land plants, such as *S. moellendorffii*, *P. patens*, *M. polymorpha*, *S. fallax*, demonstrate similar exon–intron patterns (Supplementary Figure S5). The conserved exon–intron structure further supported a single origin of subgroup III genes.

### Stepwise Expansion of the R2R3-MYB Genes During Early Evolutionary Stage of Land Plants and Angiosperms

**FIGURE 3:**
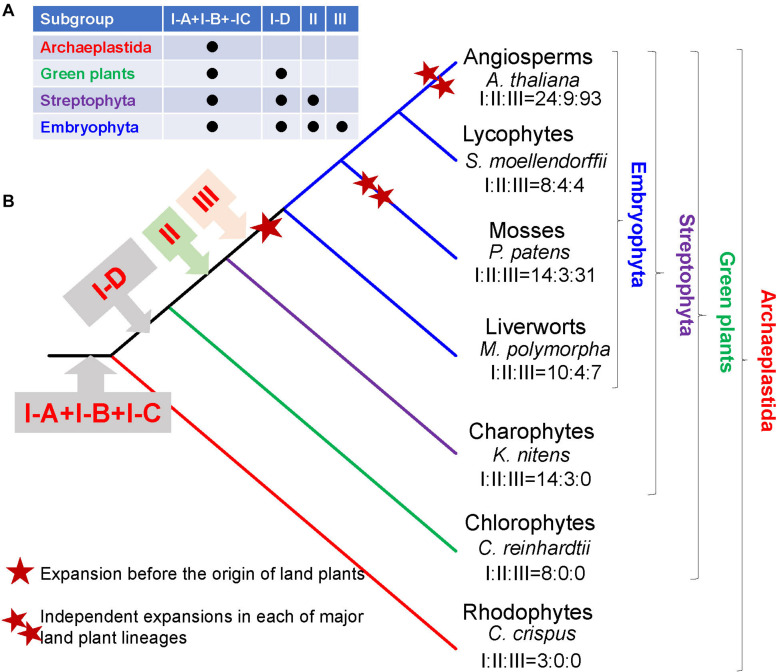
Schematic illustration of stepwise expansion of R2R3-MYB gene family in plants. **(A)** The distribution of three subgroups (I, II, III) in different plant lineages. Subgroup I includes four clades: I-A, I-B, I-C, and I-D. **(B)** The origin of the subgroups during plant evolution. The numbers of R2R3-MYB genes in each subgroup (I, II, III) are given under the species name of representative species.

Our phylogenetic analysis showed that the R2R3-MYB gene family might have experienced two rounds of major expansions. The first major expansion is exemplified by the presence of 15 clades in subgroup III with members from one or more deep land plant lineages, supporting their formation in the very early stage of land plant evolution (Figure 2). Noticeably, most R2R3-MYB genes in land plants belong to subgroup III (Table 1). For example, 93 out of 126 (73%) R2R3-MYB genes in *Arabidopsis*, 82.9% in rice belong to the subgroup III. Therefore, most R2R3-MYB genes of subgroup III in land plants could be traced back to a single founder gene present before the divergence of land plants. The estimated number of R2R3-MYB genes of subgroup III in the common ancestor of land plants was probably 15 or possibly more, yet the numbers of R2R3-MYB genes in most extant land plants are significantly greater than 15. This suggests that R2R3-MYB genes had experienced a second round of major independent expansions in each of major land plant lineages, especially during the evolution of angiosperms (Figure 3).

## Discussion

Our analyses of gene distribution, phylogenetic relationships, and gene structures support the classification of plant R2R3-MYB genes into three subgroups. Unlike traditional gene subgroup classification, we classified R2R3-MYB genes mainly based on their evolutionary ages. Previous classifications based on monophyletic clades yielded a large number of subgroups within the R2R3-MYB gene family (Hori et al., 2014; Du et al., 2015; Nishiyama et al., 2018), what makes it difficult to understand their evolutionary history. In this study, we showed that subgroup I is the most ancient group, which was probably generated prior to the divergence of all plants. Subgroup II was formed before the divergence of land plants and charophyte algae. Subgroup III probably originated from one of the subgroup II lineages by gene duplication at least before the split between the land plants and charophytes. The data suggested that the subgroup III clades had formed prior to the earliest divergence of land plants, which occurred at least ∼400 million years ago. The strong conservation of the location and phase of introns in subgroup III genes suggests that they are maintained by functional constraints. Based on the evolutionary history of R2R3-MYB gene family and the intron–exon structure of each subgroup, the most parsimonious explanation is that the two introns were already present at the equivalent positions of the ancestral subgroup III gene. Previous studies suggested that the NAC family transcription factors play important roles in the origin of terrestrial plants (Xu et al., 2014; Maugarny-Cales et al., 2016). Similar to the subgroups III and II of R2R3-MYB genes, the NAC genes are not present in chlorophytes either. Considering that over 80% of R2R3-MYB proteins in land plants were found in the subgroups III (for example, over 80% of members in rice, Arabidopsis, and tea are distributed in subgroups III: Table 1). It is reasonable to postulate that the expansion and functional diversification of R2R3-MYB genes also play a critical role in the origin and further diversification of land plants.

The challenges faced by plants in terrestrial environments led to the evolution of many physiological mechanisms and polyploidy in many species (Becker and Marin, 2009; Becker, 2013; Delwiche and Cooper, 2015; Bowman et al., 2017; Zhao et al., 2019; Zhang et al., 2019, 2020b). The subgroup III expanded from a single ancestral gene to at least 15 copies (III-A to O) in the common ancestor of land plants showing that the R2R3-MYB genes in subgroup III had experienced a major expansion prior to the divergence of land plants. Subsequent gene duplications of the 15 members of subgroup III after diversification of land plants have further increased the number of R2R3-MYB genes in land plants. Given the multiplicity of plant-specific processes controlled by R2R3-MYB transcription factors, it was postulated that the elaboration of the R2R3-MYB family might account for some of the evolutionary innovations that have contributed to the evolution of plant diversity (Riechmann et al., 2000). Based on available functional information, at least 80% of subgroup III genes are involved in metabolisms (Dubos et al., 2010; Bowman et al., 2017; Huang et al., 2018), indicating that they play key roles in metabolic regulation. The basic helix-loop-helix (bHLH) gene families have also experienced major expansions during the transition from algae to land plants (Pires and Dolan, 2010). Similar to the R2R3-MYB genes, the bHLH genes play important roles in many different aspects in plants, including modulating secondary metabolism pathways, epidermal differentiation, and responses to environmental factors in plants (Ramsay and Glover, 2005; Castillon et al., 2007). Several MYB proteins are known to form transcription complexes with bHLH proteins in plants (Ramsay and Glover, 2005), indicating that they together may play an important role in the origin of land plants. It was reported that a *cis*-regulatory mutation in an R2R3-MYB transcription factor, which is an ortholog of ATMYB113 in group III-C, results in differential regulation of enzymes in the anthocyanin biosynthetic pathway and is the major contributor to differences in floral pigmentation (Streisfeld et al., 2013). The expansions of R2R3-MYB genes prior to the diversification of land plants and the subsequent modifications of *cis*-regulatory elements, might have provided more genetic materials for the adaptation of ancestral land plants to terrestrial environments.

## Data Availability Statement

The datasets presented in this study can be found in online repositories. The names of the repository/repositories and accession number(s) can be found in the article/Supplementary Material.

## Author Contributions

LZ and ZGL conceived the study. XC, LZ, LW, SX, and ZLL performed the analyses. LZ and XC wrote the manuscript. Z-HC, FC, SX, and ZXL revised the manuscript and contributed to discussion. All authors read and approved the final manuscript.

## Conflict of Interest

The authors declare that the research was conducted in the absence of any commercial or financial relationships that could be construed as a potential conflict of interest.

## Footnotes

1 https://db.cngb.org/onekp/

2 https://genome.jgi.doe.gov/portal/

3 http://www.phytozome.net/

4 https://bigd.big.ac.cn/gwh/

5 https://db.cngb.org/onekp/

6 http://hmmer.janelia.org/

7 http://www.microbesonline.org/fasttree/

## Supplementary Material

Supplementary Figure 1 Maximum likelihood tree of plant R2R3-MYB genes inferred using PhyML.

Supplementary Figure 2 Maximum likelihood tree of plant R2R3-MYB genes inferred using IQ-TREE.

Supplementary Figure 3 Maximum likelihood tree of plant R2R3-MYB genes inferred using IQ-TREE and rooted with cdc5 genes as outgroup.

Supplementary Figure 4 Maximum likelihood tree (inferred using FastTree) and intron–exon structure of the R2R3-MYB genes in *A. thaliana.* The boxes indicate the protein-encoding exons and are drawn to scale. The introns are represented by angled lines, and the thickness of the lines is proportional to intron length (see the bottom of the figure). The core sequence is marked in blue in the exons.

Supplementary Figure 5 The intron–exon structure of R2R3-MYB genes from *Selaginella moellendorffii*, *Physcomitrella patens*, *Marchantia polymorpha*, and *Sphagnum fallax*.

Supplementary Table 1 Gene list of R2R3-MYB genes in each group in each species from genome and transcriptomes.

Supplementary File 1 Amino acid sequences of R2R3-MYB genes used in this study.

Supplementary File 2 Multiple sequence alignment used to infer the phylogenetic tree in Figure 1 and Supplementary Figures S1, S2.

Supplementary File 3 Multiple sequence alignment used to infer the phylogenetic tree in Supplementary Figure S3.
