# Supplementary material for: Origins and Stepwise Expansion of R2R3-MYB Transcription Factors for the Terrestrial Adaptation of Plants
Source: Front Plant Sci. 2020 Dec 23;11:575360. doi: 10.3389/fpls.2020.575360 (PMC7785938; doi:10.3389/fpls.2020.575360)
Supplement: Supplementary Figure 1 — Maximum likelihood tree of plant R2R3-MYB genes inferred using PhyML. [file Data_Sheet_1.PDF]

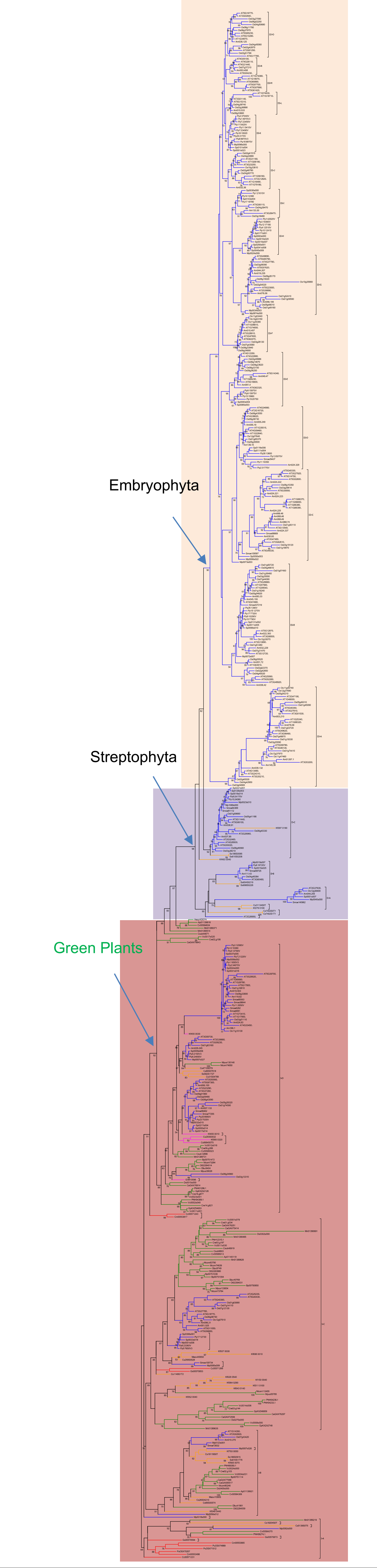

Embryophyta
Streptophyta
Green Plants

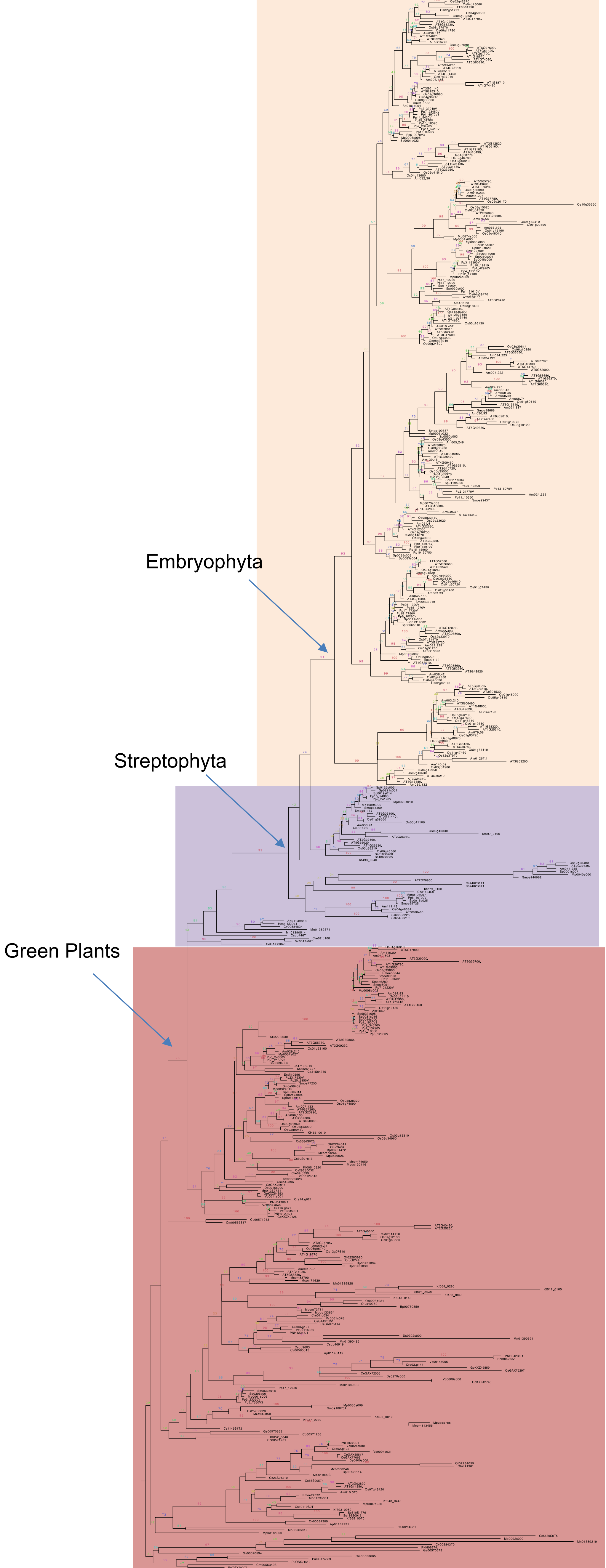

Embryophyta
Streptophyta
Green Plants

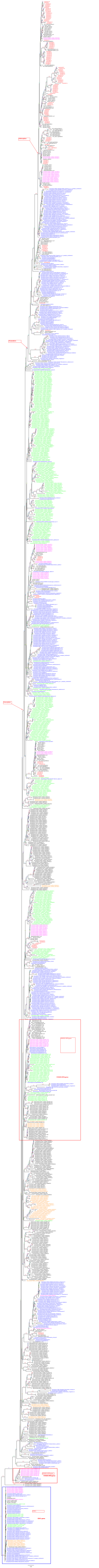

R1R2R3-MYB genes
CDC5
CDC5 genes
0.8

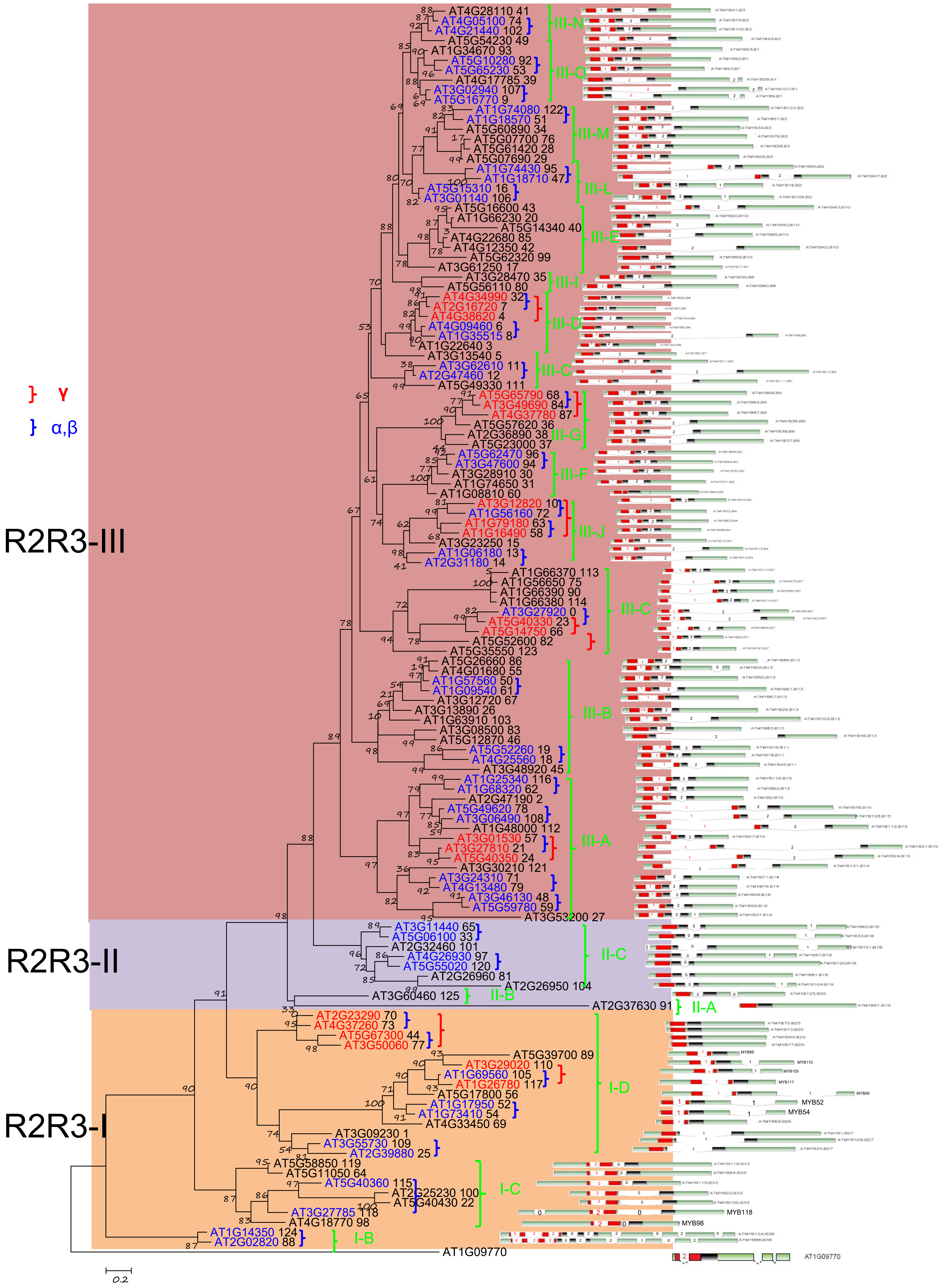

R2R3-III
R2R3-II
R2R3-I
γ
α,β
AT4G28110 41
AT4G05100 74
AT4G21440 102
III-N
AT5G54230 49
AT1G34670 93
AT5G10280 92
AT5G65230 53
III-O
AT4G17785 39
AT3G02940 107
AT5G16770 9
AT1G74080 122
AT1G18570 51
AT5G60890 34
III-M
AT5G07700 76
AT5G61420 28
AT5G07690 29
AT1G74430 95
AT1G18710 47
III-L
AT5G15310 16
AT3G01140 106
AT5G16600 43
AT1G66230 20
AT5G14340 40
III-E
AT4G22680 85
AT4G12350 42
AT5G62320 99
AT3G61250 17
AT3G28470 35
III-I
AT5G56110 80
AT4G34990 32
AT2G16720 7
AT4G38620 4
III-D
AT4G09460 6
AT1G35515 8
AT1G22640 3
AT3G13540 5
AT3G62610 11
III-C
AT2G47460 12
AT5G49330 111
AT5G65790 68
AT3G49690 84
AT4G37780 87
AT5G57620 36
AT2G36890 38
III-G
AT5G23000 37
AT5G62470 96
AT3G47600 94
III-F
AT3G28910 30
AT1G74650 31
AT1G08810 60
AT3G12820 10
AT1G56160 72
AT1G79180 63
III-J
AT1G16490 58
AT3G23250 15
AT1G06180 13
AT2G31180 14
AT1G66370 113
AT1G56650 75
AT1G66390 90
AT1G66380 114
III-C
AT3G27920 0
AT5G40330 23
AT5G14750 66
AT5G52600 82
AT5G35550 123
AT5G26660 86
AT4G01680 55
AT1G57560 50
AT1G09540 61
AT3G12720 67
AT3G13890 26
AT1G63910 103
III-B
AT3G08500 83
AT5G12870 46
AT5G52260 19
AT4G25560 18
AT3G48920 45
AT1G25340 116
AT1G68320 62
AT2G47190 2
AT5G49620 78
AT3G06490 108
AT1G48000 112
III-A
AT3G01530 57
AT3G27810 21
AT5G40350 24
AT3G30210 121
AT3G24310 71
AT4G13480 79
AT3G46130 48
AT5G59780 59
AT3G53200 27
AT3G11440 65
AT5G06100 33
AT2G32460 101
II-C
AT4G26930 97
AT5G55020 120
AT2G26960 81
AT2G26950 104
AT3G60460 125
II-B
AT2G37630 91
II-A
AT2G23290 70
AT4G37260 73
AT5G67300 44
AT3G50060 77
AT5G39700 89
AT3G29020 110
AT1G69560 105
AT1G26780 117
AT5G17800 56
I-D
AT1G17950 52
AT1G73410 54
AT4G33450 69
AT3G09230 1
AT3G55730 109
AT2G39880 25
AT5G58850 119
AT5G11050 64
AT5G40360 115
AT2G25230 100
I-C
AT5G40430 22
AT3G27785 118
AT4G18770 98
AT1G14350 124
AT2G02820 88
I-B
AT1G09770
0.2
MYB89
MYB110
MYB105
MYB117
MYB56
MYB52
MYB54
MYB118
MYB98
AT1G09770

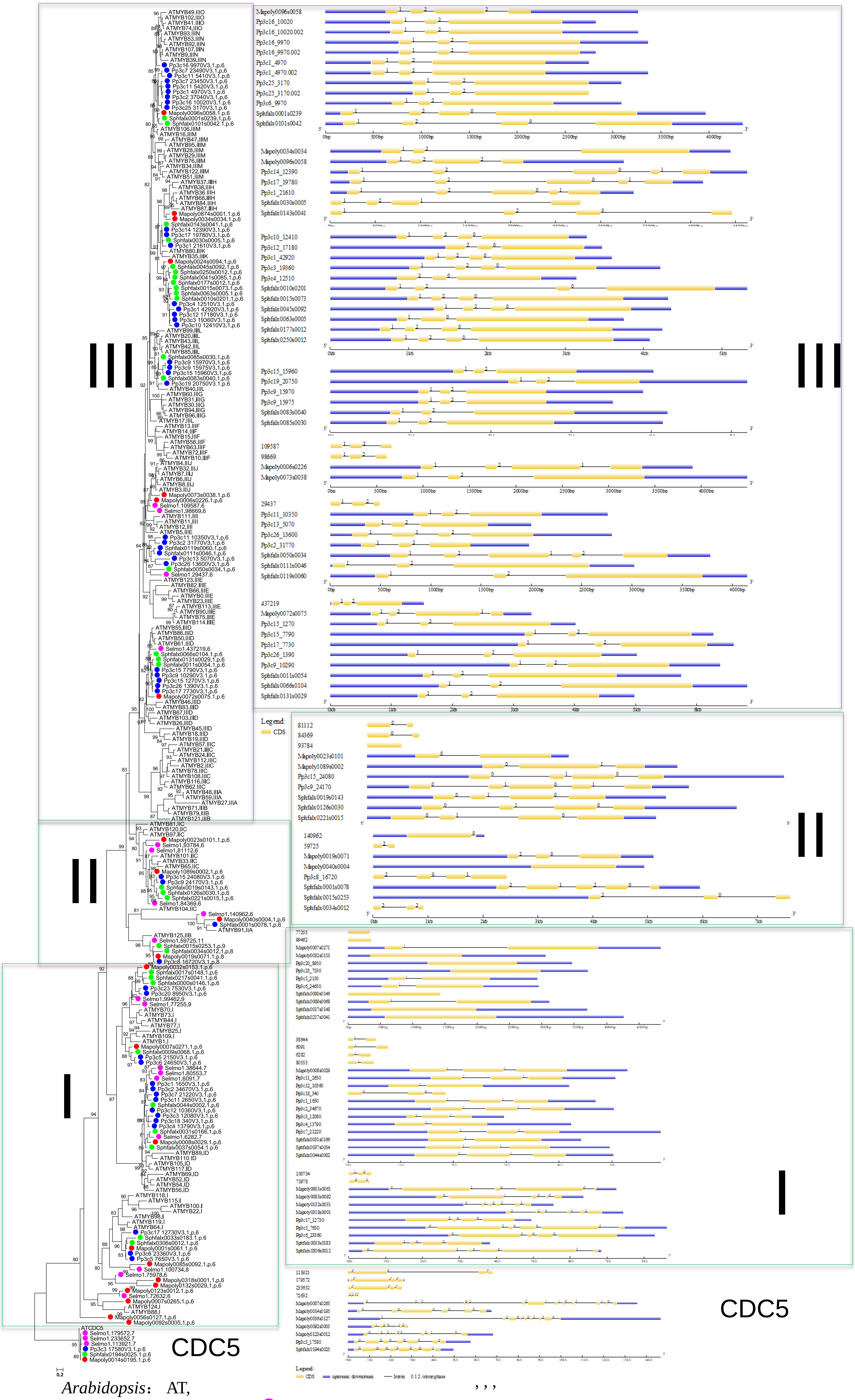

Arabidopsis: AT,

S. moellendorffii: Selmo1

P. patens: Pp3C

S. Fallax: Sphfal

M. polymorpha: Mapoly
